# Effect of a probiotic preparation (VSL#3) in critically ill patients: A randomized, double-blind, placebo-controlled trial (Pilot Study)

**DOI:** 10.12669/pjms.292.3370

**Published:** 2013-04

**Authors:** Mehrangiz Ebrahimi-Mameghani, Sarvin Sanaie, Ata Mahmoodpoor, Hadi Hamishehkar

**Affiliations:** 1 Mehrangiz Ebrahimi-Mameghani, Associated Professor of Nutrition, Nutrition Research Center, Faculty of Nutrition, Tabriz University of Medical Sciences, Tabriz, Iran.; 2 Sarvin Sanaie, PhD Student of Nutrition, MD, Student Research Center, Faculty of Nutrition, Tabriz University of Medical Sciences, Tabriz, Iran.; 3 Ata Mahmoodpoor, Assistant Professor of Anesthesiology, Fellowship of Critical Care Medicine, Anesthesiology and Intensive Care Department, Faculty of Medicine, Tabriz University of Medical Sciences, Tabriz, Iran.; 4 Hadi Hamishehkar, Assistant Professor of Clinical Pharmacy, Clinical Pharmacy Department, Faculty of Pharmacy, Tabriz University of Medical Sciences, Tabriz, Iran.

**Keywords:** Probiotic, Critically ill, Oxidative stress, Sepsis

## Abstract

***Objective*** ***: ***Reactive oxygen species (ROS) are a major contributing factor in diseases pathophysiology in critically ill patients. Oxidative stress usually occurs in critical illnesses, specifically during sepsis, and organ dysfunction. The anti-oxidative properties of probiotics may serve as a defense in intestine and overcome various oxidative stresses. The aim of this trial was to determine the effect of probiotics on inflammation, antioxidant capacity and lipid peroxidation in critically ill patients**.**

***Methodology*** ***:*** Forty patients admitted to the intensive care unit were enrolled in this double-blind, randomized controlled trial. They were randomized to receive placebo or probiotic for 7 days. Serum levels of Total Antioxidant Capacity (TAC), Malodialdehyde (MDA), C-Reactive Protein (CRP) and Acute Physiology and Chronic Health Evaluation (APACHE II) score were measured before initiation of the study and on the 7^th^ day.

***Results: ***There was a significant difference in CRP levels and APACHE II score between two groups at the end of the study (*P*= 0.003 and 0.001, respectively). There was not a significant difference in levels of TAC and MDA between two groups.

***Conclusions: ***Administration of probiotics to critically ill patients caused reduction in inflammation and improvement of clinical outcome. However, there were not significant changes in markers of oxidative stress.

## INTRODUCTION

Reactive oxygen species (ROS) are a major contributing factor of diseases in critically ill patients.^1^ Oxidative stress is the imbalance between ROS production and the body’s defense system. Scavengers of ROS and antioxidants are important in treating and preventing the damage caused by oxidative stress.^2^^,^^3^ Critically ill patients can have increased level of ROS or decreased antioxidant defenses. Several studies show that oxidative stress occurs in critical illnesses, specifically in patients with sepsis and organ dysfunction^4^^,^^5^ as ROS can stimulate the inflammatory system. Critical illness is associated with massive oxidative stress resulting in exacerbation of organ injury and poor clinical outcome.^6^ The severity of illness by APACHE III has been proportionally related to the degree of oxidative stress.^7^ Sepsis and multiple organ dysfunction syndrome (MODS) are the most common cause of death in critically ill patients.^8^ Destruction of intestinal barrier function and increased translocation of bacteria or their toxins into systemic blood flow can lead to risk of infection and MODS in critically ill patients.^9^^,^^10^

Nowadays the role of inflammation and oxidative stress in the pathogenesis of sepsis is obvious.^11^ ROS generation plays a key role in survival for septic patients.^12^Numerous studies have reported severe oxidative stress in patients with SIRS and sepsis, with reduced total antioxidant capacity (TAC)^13^^,^^14^ and increased levels of Thiobarbituric acid reactive substances^15^ and malondialdehyde (MDA).^14^^,^^15^ TAC gives information about all of the antioxidants in the organism, while MDA is a lipid peroxidation marker used to assess lipid peroxidation due to increased oxidative stress.^16^

Various therapies including antioxidants especially selenium^17^^,^^18^ have been evaluated in sepsis but none of them has been effective on survival of patients.^19^ Probiotics are “live microorganisms which when administered in adequate amounts confer a health benefit on the host”.^20^ The antioxidant effects of probiotics have been reported in different studies.^21^^-^^23^ Their anti-oxidative properties may serve as a defense in intestine and overcome various oxidative stresses^23^, so they can prevent or control several disease associated with oxidative stress^24^ and they seem to have beneficial effects in improvement of critically ill patients.

The purpose of this randomized clinical trial was to determine the effect of probiotic containing lactobacillus, bifidobacterium and streptococcus thermophiluson inflammation, antioxidant capacity and lipid peroxidation in critically ill patients in the intensive care unit.

## METHODOLOGY

Patients admitted between December of 2011 and October of 2012 to the ICU of the Shohada Hospital (Tabriz, East Azerbaijan, Iran) were eligible for the study. After approval of ethics committee of Tabriz University of Medical Sciences and obtaining informed consent of the patients or their legal guardian, 40 patients were enrolled in this trial. The clinical trial of the study was registered in Iranian Registry of Clinical Trials with code number of (IRCT201112143320N6).

This is a report of the data base from PhD thesis entitled “Effect of probiotic containing lactobacillus, bifidobacterium and streptococusthermophilus administration on inflammatory, coagulation and oxidative factors in critically ill patients with risk of sepsis”. A total sample size of 40 subjects was calculated based on the published levels of IL-6 differences in critically ill patients^25^ at the 5% significance level with a power of 80%.The formula used for sample size calculation was as: n=[2(SD^2^) (Z _α/2 _+ Z _β _) ^2^] / ∆^2^ , which SD was 50 and ∆ was 50. Using the formula, a sample size of 15.7 for each group was achieved which regarding the loss to follow, we included 20 patients in each group.

Inclusion criteria were critically ill patients admitted to the surgical ICU with positive SIRS and APACHE II score of 15 to 30, aging 18 to 40 years old and receiving enteral nutrition who were expected to stay in ICU for at least 7 days. Exclusion criteria were pregnant and lactating women, patients who cannot tolerate enteral nutrition, those with unstable hemodynamic, immune disorders, intestinal obstruction or ischemia, cancer and patients who were expected to expire in the next 24 hours. All patients received enteral nutrition with *Fresubin original fibre (Fresenius Kabi, Homburg, *Germany) at the first 24-hours of the admission via nasogastric tube. It was started at 25mL/h and increased by 25 mL/h every 4 h until the target rate was achieved. Weight and height of the patients were recorded and body mass index was calculated by the formula weight (in kg)/height^2^ (in m). Energy requirements were calculated as 25–30 kcal/kg and protein requirements as 1.2–1.5 g/kg.

The patients were randomly assigned into two 20-person groups; the first group received standard treatment plus placebo and the second group received standard treatment plus VSL#3 (VSL Pharmaceuticals, Ft Lauderdale, FL), 2 sachets daily for 7 days. Each sachet of probiotics contained 900 billion viable lyophilized bacteria consisting of 4 strains of *Lactobacillus *(*L. casei, L. plantarum, L. acidophilus, *and *L. delbrueckii*subsp. *Bulgaricus*), 3 strains of *Bifidobacterium *(*B. longum, B. breve,* and *B. infantis*) and *Streptococcus salivarius *subsp. *Thermophilus*. Blood was obtained from each patient before the study and on day 7 to evaluate TAC, MDA and CRP. TAC levels were measured using an ELISA kit (ImAnOx, Immundiagnoctic AG, Bensheim, Germany) and MDA levels were measured based on reaction with t*hiobarbituric acid and a s*pectrophotometric Assay. APACHE II score was also determined for all patients at baseline and on the 7^th^ day. Data were analyzed by SPSS 16.* P *values<0.05 were considered significant for all statistical tests.

## RESULTS

**Fig.1:**
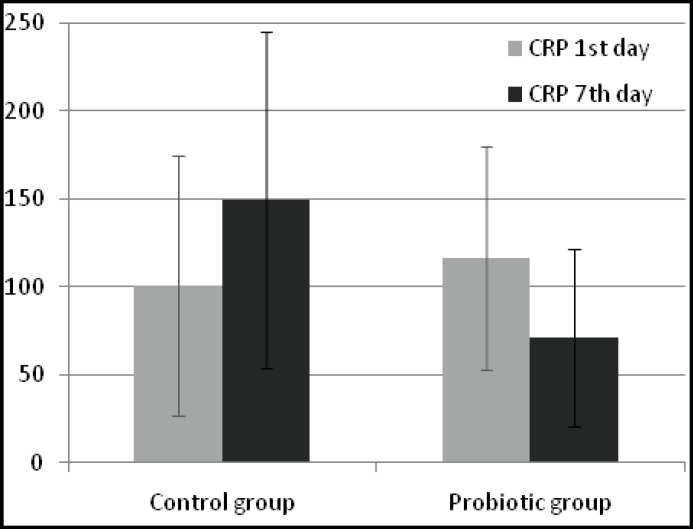
Mean (±SD) CRP levels in two groups of patients. *Patients in the probiotic group had significantly lower CRP levels by day 7 (*P* =0.006

There were 20 patients in probiotic group and 20 patients in control group. No significant differences in demographic data of patients were observed between two groups (Table-I). Levels of TAC, MDA, CRP and APACHE II at the baseline and at the end of the study are shown in Table-II.TAC has significantly increased and MDA has significantly decreased in both groups, however there isn't any significant difference in their levels between two groups after the treatment (*P*=0.062 and 0.123, respectively). Levels of CRP (Fig.1) and APACHE II score were significantly lower in the probiotic group compared to controls at day 7 (*P*=0.003 and 0.001, respectively).

**Table-I:** Demographic data of patients

	*Control group *	*Probiotic group*	*P value*
Age (year)	35.60±5.03^*^	33.60±5.50	0.238
Male/female	14/6	13/7	0.500
BMI	24.70±3	24.30±2.92	0.677

^*^mean±SD           BMI: Body Mass Index

**Table-II:** Levels of TAC, MDA, CRP and APACHE II at baseline and at the end of the study

		*Control group*	*Probiotic group*	*P value*
TAC(μmol/L)	1^st^ day	122.43±53.50*	96.67±38.21	0.088^†^
	7^th^ day	153.55±73.25	191.95±51.28	0.062
MDA(nmol/ml)	1^st^ day	3.28±1.58	4.02±1.64	0.159
	7^th^ day	2.52±1.19	1.90±1.29	0.123
CRP(mg/l)	1^st^ day	100.57±73.81	116.38±63.25	0.471
	7^th^ day	149.56±95.83	71.20±50.43	0.003
APACHE	1^st^ day	22.45±4.57	22.80±4.73	0.813
	7^th^ day	20.85±7.55	13.85±4.82	0.001

^*^mean±SD           ^†^Independent-samples T test

## DISCUSSION

The present study used a double-blind, placebo-controlled, randomized design to determine the effects of probiotics on markers of oxidative stress and inflammation in critically ill, enterally fed patients. Overall, the patients who received probiotic showed a greater reduction in inflammation than did the patients who received placebo.CRP is an acute-phase protein produced by the liver and by endothelial cells.^26^ It is commonly used as a marker of systemic inflammation^27^ and serum levels provide a useful indicator of the extent of an inflammatory process.^28^ CRP inhibits the production of proinflammatory cytokines and chemokines.^29^ In the present study patients receiving probiotic showed a reduction in CRP concentrations over the treatment. A similar finding was reported by Kotzampassi et al, who showed that a combination regimen of pro- and prebiotics (Synbiotic 2000 Forte) caused a significant reduction in CRP levels compared to placebo-treated group.^30^ Interestingly, in a study by Alberda et al which critically ill patients were assigned to receive viable probiotics (VSL#3), equivalent probiotic sonicates or placebo, although most of the patients showed a reduction in CRP concentrations over the treatment period, patients receiving viable probiotics had a lesser decline in CRP concentrations than patients receiving either placebo or bacterial sonicates.^31^

In another study by McNaught et al, enteral administration of ProViva, an oatmeal-based drink containing *Lactobacillus plantarum *299v to critically ill patients had no significant effect on serum CRP levels.^25^ The type of probiotic used in the studies may be an explanation for these dissimilar findings.VSL#3 is a potent probiotic medical food that delivers the highest available concentration of beneficial live bacteria of any other probiotic and contains 8 different strains of live lactic acid bacteria specially selected to produce an optimal synergistic composition of bacteria.^32^^,^^33^ Although the type of the probiotic used in the study of Alberda et al^30^ was the same as the one we used and even duration of the treatment was the same in both studies (7 days), the different results seen for the CRP can be due to the patients' population. Our trial was performed in a surgical ICU which most of them were traumatic patients, so heterogeneity which is one the most important problems in ICU population was decreased.

APACHE II score which is a measurement of disease severity is closely correlated with the risk of many common diseases and hospital death.^34^ In our study, APACHE II score significantly decreased in probiotic group after 7 days of treatment. However, in a study performed on severe traumatic brain-injured patients with Glasgow Coma Scale scores between 5 and 8, APACHE score was not significantly affected by probiotic treatment.^35^The improvement in clinical outcome shown by a significant decrease in APACHE II score in this study can be related to the reduction of inflammation by the use of probiotics.

Of the many biological targets of oxidative stress, lipids are the most involved class of bio-molecules. Lipid oxidation gives rise to a number of secondary products. Malondialdehyde (MDA) is the principal and most studied product of polyunsaturated fatty acid peroxidation.^36^ TAC had a significant increase and MDA had a significant decrease in both groups at the end of the study, which can be due to the usage of the various antioxidant drugs in the standard treatment of the patients in intensive care unit. Nevertheless, there was not a significant difference between two groups after 7 days of treatment with probiotics. However, the increase of TAC is more in probiotic group compared to control group which shows that body's antioxidant capacity increases with the use of probiotics and according to the *P* value (*P*= 0.062), it seems that significant results could be achieved by increasing sample size. Although different studies have shown the anti-oxidative effects of probiotics^21^^-^^24^, to the best of our knowledge, no study has been performed regarding the effect of probiotic administration on markers of oxidative stress in critically ill patients.

***Limitation of the study:*** This is a single center study with 40 patients included, so we need future multi center trials with larger sample size of critically ill patients. This trial was performed in surgical patients, so for routine usage of probiotics in critically ill patients, they should be examined in medical or mixed type ICUs. There is also need to trials that define the best dosage and optimal duration of therapy in these patients.

## CONCLUSION

In conclusion, the results of this randomized trial suggest that administration of probiotics in critically ill patients is associated with clinical benefits compared to placebo-treated patients: they significantly reduce the levels of CRP and APACHE score. However, they did not significantly affect the levels of the markers of oxidative stress. So, further studies with larger sample size are needed to clarify their usefulness in this group of patients. 

## Authors' contribution:

ME: Study design, revising manuscript. SS: Study design, sample collection and preparing manuscript draft. AM: Study design, sample collection and revising manuscript. HH: Sample analyzing via commercially available enzyme-linked immunosorbent assay kit.
